# The *Brucella abortus* two-component system response regulator BvrR binds to three DNA regulatory boxes in the upstream region of *omp25*

**DOI:** 10.3389/fmicb.2023.1241143

**Published:** 2023-09-14

**Authors:** Amanda Castillo-Zeledón, Olga Rivas-Solano, Fabián Villalta-Romero, Olman Gómez-Espinoza, Edgardo Moreno, Esteban Chaves-Olarte, Caterina Guzmán-Verri

**Affiliations:** ^1^Programa de Investigación en Enfermedades Tropicales, Escuela de Medicina Veterinaria, Universidad Nacional de Costa Rica, Heredia, Costa Rica; ^2^Centro de Investigación en Biotecnología, Escuela de Biología, Instituto Tecnológico de Costa Rica, Campus Tecnológico Central Cartago, Cartago, Costa Rica; ^3^Centro de Investigación en Enfermedades Tropicales, Facultad de Microbiología, Universidad de Costa Rica, San José, Costa Rica

**Keywords:** two-component regulatory system, outer membrane protein (OMP), *Rhizobiales*, *Brucella*, *Brucella abortus*

## Abstract

*Brucella abortus* is a facultative extracellular-intracellular bacterial zoonotic pathogen worldwide. It is also a major cause of abortion in bovines, generating economic losses. The two-component regulatory system BvrR/BvrS modulates the expression of genes required to transition from extracellular to intracellular lifestyles. However, few regulatory regions of BvrR direct target genes have been studied. In this study, we characterized the regulatory region of *omp25*, a gene encoding an outer membrane protein that is positively regulated by TCS BvrR/BvrS. By *omp25*-*lacZ* reporter fusions and β-galactosidase activity assays, we found that the region between-262 and + 127 is necessary for transcriptional activity, particularly a 111-bp long fragment located from-262 to −152. In addition, we demonstrated the binding of P-BvrR to three sites within the −140 to +1 region. Two of these sites were delimited between −18 to +1 and − 99 to −76 by DNase I footprinting and called DNA regulatory boxes 1 and 2, respectively. The third binding site (box 3) was delimited from −140 to −122 by combining EMSA and fluorescence anisotropy results. A molecular docking analysis with HDOCK predicted BvrR-DNA interactions between 11, 13, and 12 amino acid residue-nucleotide pairs in boxes 1, 2, and 3, respectively. A manual sequence alignment of the three regulatory boxes revealed the presence of inverted and non-inverted repeats of five to eight nucleotides, partially matching DNA binding motifs previously described for BvrR. We propose that P-BvrR binds directly to up to three regulatory boxes and probably interacts with other transcription factors to regulate *omp25* expression. This gene regulation model could apply to other BvrR target genes and to orthologs of the TCS BvrR/BvrS and Omp25 in phylogenetically closed *Rhizobiales*.

## 1. Introduction

*Brucella abortus* is a facultative extracellular-intracellular Gram-negative pathogen. It belongs to *Rhizobiales*, an order composed of cell-associated pathogens, symbionts, and free-living bacteria (Batut et al., 2004; Moreno, 2021). *B. abortus* causes brucellosis, a widely distributed zoonotic disease. In infected cattle, the disease manifests with abortion and infertility, causing economic losses (Spink, 1957).

The pathogenicity of brucellae resides in their ability to invade, survive, and replicate inside host cells (Roop et al., 2021). In *B. abortus*, the two-component regulatory system (TCS), BvrR/BvrS, is important for the transition from the extracellular to the intracellular milieu (Sola-Landa et al., 1998; Guzman-Verri et al., 2002; López-Goñi et al., 2002; Altamirano-Silva et al., 2018). This TCS comprises a transmembrane sensor protein with histidine kinase activity called BvrS and a cytoplasmic response regulator called BvrR, which has homology to OmpR (López-Goñi et al., 2002; Altamirano-Silva et al., 2018).

Phylogenetic analyses revealed that the TCS BvrR/BvrS is orthologous to other *Rhizobiales* TCSs, including ExoS/ChvI from the plant endosymbiont *Sinorhizobium meliloti*, ChvG/ChvI from the plant pericellular pathogen *Agrobacterium tumefaciens*, and BatR/BatS from the intracellular zoonotic pathogen *Bartonella* sp. Those orthologous TCSs respond to environmental conditions and regulate the expression of target genes involved in distinct stages of host invasion and intracellular survival (Charles and Nester, 1993; Cheng and Walker, 1998; Batut et al., 2004; Beier and Gross, 2006; Quebatte et al., 2010; Bélanger and Charles, 2013; Heavner et al., 2015; Ratib et al., 2018).

In brucellae, BvrS senses low pH and low nutrient availability, conditions probably encountered when the bacterium is trafficking through the endosomal pathway (Altamirano-Silva et al., 2018, 2021). Following this, BvrS probably auto-phosphorylates and transduces the signal via a phosphate group to BvrR, increasing its affinity for specific chromosomal regions (López-Goñi et al., 2002; Nguyen et al., 2015; Altamirano-Silva et al., 2021). A *B. abortus bvrR* mutant lacks virulence in murine models and does not replicate in cell culture models (Sola-Landa et al., 1998). This mutant differentially expresses outer membrane and periplasmic proteins (Guzman-Verri et al., 2002; Lamontagne et al., 2007; Viadas et al., 2010) and shows a distinctive lipopolysaccharide acylation pattern compared to the wild-type strain (Manterola et al., 2005). Regarding outer membrane proteins, the TCS BvrR/BvrS positively regulates the expression of *omp25* (Guzman-Verri et al., 2002; Lamontagne et al., 2007; Viadas et al., 2010). This gene encodes a major outer-membrane protein of 25 kDa (Omp25) belonging to the Omp25/31 family (Vizcaíno et al., 2001), the most abundant outer-membrane proteins of brucellae (Martín-Martín et al., 2009). In *B. abortus*, although Omp25 is not essential for the invasion, survival, and replication inside RAW macrophages and HeLa cells, it has a structural function in the covalent attachment of the outer membrane to peptidoglycan (Manterola et al., 2007; Godessart et al., 2021).

The TCS BvrR/BvrS also regulates the expression of virulence genes related to intracellular trafficking and cell egress, like the Type IV Secretion System VirB and the quorum-sensing regulator VjbR (Lamontagne et al., 2009; Martínez-Núñez et al., 2010; Viadas et al., 2010; Altamirano-Silva et al., 2018, 2021), and is related to the carbon and nitrogen metabolic fitness according to the encountered environment (Lamontagne et al., 2009; Viadas et al., 2010; Rivas-Solano et al., 2022).

Recently, two DNA binding motifs putatively recognized by BvrR have been reported by *in silico* predictions (Ramírez-González et al., 2019) and experimental approaches (Rivas-Solano et al., 2022).

A direct interaction has been described between BvrR and the upstream region of *omp25*, located between coordinates −159 and + 34 from the start codon (Rivas-Solano et al., 2022). Two transcriptional start sites (TSS) have been independently reported for *omp25*, at positions −131 and *−* 82 (Suárez-Esquivel et al., 2016; Rivas-Solano et al., 2022).

Here, we characterized the *omp25* transcriptional regulatory region as a prototype of a regulatory element directly controlled by the TCS BvrR/BvrS. Our results suggest that the TCS BvrR/BvrS regulates *omp25* expression directly by binding to up to three regulatory boxes with inverted and non-inverted DNA repeats. The research presented here contributes to understanding how the TCS BvrR/BvrS regulates target genes and might apply to other ortholog TCSs in *Rhizobiales*.

## 2. Materials and methods

### 2.1. Bacterial strains and culture conditions

*Escherichia coli* and *B. abortus* strains (Table 1) were incubated at 37°C at 200 rpm and grown on Luria Bertani Broth (LB) (Sambrook et al., 1989) or Tryptic Soy Broth (TSB) (Suárez-Esquivel et al., 2016). Additionally, culture media were supplemented with antibiotics (kanamycin 30 μg/ml, gentamicin 20 μg/ml, or ampicillin 100 μg/ml) when necessary. All procedures involving live *B. abortus* were performed according to the “Reglamento de Bioseguridad de la CCSS 39975–0,” 2012, after the “Decreto Ejecutivo #30965-S,” 2002, and research protocol SIA 0652-19, approved by the National University, Costa Rica.

**Table 1:** Bacterial strains and plasmids.

Strain	Relevant characteristics	Reference
*E. coli*		
XL1Blue	*recA1 endA1 gyrA96 thi-1 hsdR17 supE44 relA1 Δ(lac-proAB) [*F′ *proAB lacIqZΔM15].* Tn*10(*Tet′*)*	Sambrook et al. (1989)
E392	XL1Blue carrier of p392, Km^r^	This study
E262	XL1Blue carrier of p262, Km^r^	This study
E151	XL1Blue carrier of p151, Km^r^	This study
*B. abortus*		
2308 W	Wild-type, virulent strain, NaI^r^	Suárez-Esquivel et al. (2016)
3aZ	2308 W carrier of transcripcional chromosomal fusion P*omp3a*::*lacZ*, Gm^r^ Amp^r^	Guzman-Verri et al. (2002)
B392	2308 W carrier of p392, Km^r^	This study
B262	2308 W carrier of p262, Km^r^	This study
B151	2308 W carrier of p151, Km^r^	This study
BpMR15	2308 W carrier of pMR15, Kmr	This study
Plasmids		
pMR15	High copy number vector, promoterless *lacZ* gene, Km^r^	Gober and Shapiro (1992), Courtesy of M. Roop.
p392	pMR15-derivative with a cloned fragment of 521-bp, 392-bp upstream *omp25*, and the first 127-bp of the coding sequence, Km^r^	This study
p262	pMR15-derivative with a cloned fragment of 391-bp, 262-bp upstream *omp25*, and the first 127-bp of the coding sequence, Km^r^	This study
p151	pMR15-derivative with a cloned fragment of 280-bp, 151-bp upstream *omp25*, and the first 127-bp of the coding sequence, Km^r^	This study

Nal^r^, resistance to nalidixic acid; Gm^r^, resistance to gentamicin; Amp^r^, resistance to ampicillin; Km^r^, resistance to kanamycin.

### 2.2. Construction of transcriptional fusions and β-galactosidase activity assays

**Figure 1:**
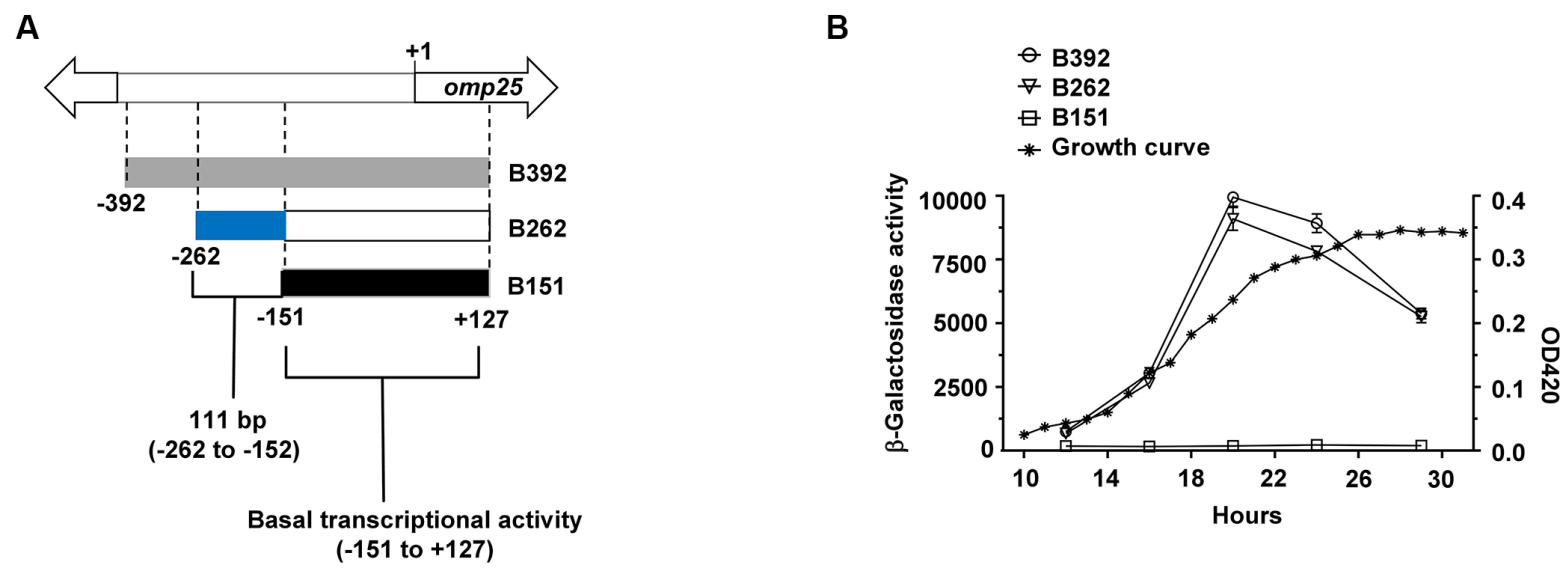
A 111-bp fragment upstream of *omp25* is needed for wild-type levels of transcription. **(A)** Schematic representation of the *omp25* upstream region analyzed by constructing three β-galactosidase transcriptional fusions. Gray rectangle = region with wild-type levels of transcription (from −392 to +127), blue rectangle = 111-bp region (from −262 to −152) needed for wild-type levels of transcription, and black rectangle = fragment with basal transcriptional activity. DNA coordinates are given according to the *omp25* adenine in the start codon. **(B)** β-galactosidase results (unfilled figures) and a representative growth curve (asterisks). *B. abortus* 2308 W-derived strains containing the promoterless reporter vector fusions with upstream *omp25* fragments were grown in TSB at 37°C and assayed for β-galactosidase activity at different times of the growth curve. Absorbance was measured at 420 nm at the indicated times. B151 presented significant statistical differences for β-galactosidase activity compared to the rest of the strains. These results are representative of at least three independent experiments. The residual β-galactosidase activity from the empty vector (BpMR15) was removed from each test carried out in the corresponding growth phases (one-way ANOVA followed by Tukey’s multiple comparisons test) (*p* < 0.05).

The primers used in this study are detailed in Supplementary Table 1. A DNA fragment from the genome of *B. abortus* 2308 W (GenBank Accession ERS568782), encompassing the *omp25* region from −392 to +127 and two smaller ones from −262 to +127 and −151 to +127 (Figure 1A), was amplified by PCR and purified with the QIAquick® Gel Extraction Kit (Qiagen). The amplicons and the pMR15 vector (Table 1) were excised separately with BamHI (10 U/μl) and XbaI (10 U/μl) (Fermentas®) for 18 h at 37°C. The restriction enzymes were inactivated at 80°C for 20 min. The DNA fragments were ligated with the pMR15 vector using T4 DNA ligase (5 U/μl) (Fermentas®) at room temperature overnight to obtain plasmids p392, p262, and p151 (Table 1). Then, the plasmids were electroporated into the *E. coli* strain XL1-Blue to generate the strains E392, E262, and E151 (Table 1) using the Electro Cell Manipulator ECM 630 BTX®. Colonies with the new plasmids were selected using kanamycin and screened using primers omp25lacZF and omp25lacZR (Supplementary Table 1). Plasmid DNA was isolated and electroporated into *B. abortus* using the Electro Cell Manipulator ECM 630 BTX® to obtain the strains B392, B262, and B151 (Table 1). The vector pMR15 was also electroporated into *B. abortus* as a non-promoter activity control (strain BpMR15, Table 1). The β-galactosidase assays were performed with modifications (Guzman-Verri et al., 2002). Bacteria were grown until the exponential phase, permeabilized with 0.5% sodium dodecyl sulfate (SDS), 6% chloroform for 10 min at 28°C, and incubated with O-nitrophenyl-β-D-galactopyranoside (ONPG) for 10 min at 28°C. The reaction was stopped with 1 M sodium carbonate, the absorbance was measured at 420 nm, and the specific activity was expressed as nmol of O-nitrophenol produced/min × mg protein (Miller Units). The reported β-galactosidase activity was corrected according to the residual activity obtained from the empty vector strain, BpMR15. A previously constructed *lacZ*:*omp25* chromosomal fusion *B. abortus* strain (3aZ) was used as a positive control of promoter activity (Guzman-Verri et al., 2002; Table 1). A one-way ANOVA statistical analysis followed by Tukey’s multiple comparisons test was performed using GraphPad Prism version 8.00 for Windows (Graph Pad, 2019).

### 2.3. Electrophoretic mobility shift assay (EMSA)

Expression and purification of GST-BvrR were carried out as described (Martínez-Núñez et al., 2010). Before each assay, BvrR was phosphorylated with carbamoyl phosphate as described previously (Altamirano-Silva et al., 2018). DNA probes were labeled using the DIG Gel Shift Kit 2nd Generation (Roche®). Protein and DNA probes were incubated together following the protocol described in the DIG Gel Shift Kit 2nd Generation (Roche®). Protein-DNA mixtures were separated by native polyacrylamide electrophoresis at 150 V at 4°C for 1, 1.5, or 2.5 h, depending on the size of the DNA probe. The gels were electro-blotted into positively charged Nylon membranes, and the results were visualized by an enzymatic immunoassay using anti-digoxigenin-alkaline phosphatase (InvitrogenTM Electrophoretic Mobility Shift Assay Kit). The generated chemiluminescent signals were recorded on an X-ray film. Size and shape are factors that affect the electrophoretic migratory pattern of a molecule (Hellman and Fried, 2007). In our assays, the same protein is used, but the DNA fragments differ, so specific protein-DNA complexes for each DNA fragment run by EMSA were identified based on the following criteria: absence of shifted bands in the lane without P-BvrR, absence of shifted bands in the specificity binding control, P-BvrR concentration-dependent shifted bands, and consistency between independent replicas. If any of these criteria were not met, the shifted band was classified as unspecific (Hellman and Fried, 2007; Altamirano-Silva et al., 2018).

For direct EMSA, two DNA fragments used as probes were obtained by PCR using the following primer pairs: omp25.262 and omp25.152 or omp25.262 and omp25.122 (Supplementary Table 1). Labeled probes at 0.033 μM were incubated with increasing concentrations of phosphorylated BvrR (P-BvrR) (0.1–1.6 μM) for 15 min on ice. Mixtures were electrophoresed for 2.5 h and analyzed as described above. A coding region of the ribosomal protein (*rpIL*) was amplified by PCR with the primers L12.F and L12.R (Supplementary Table 1) and used as a specificity-binding control.

For competitive EMSA, a 193-bp fragment (coordinates −159 to +34 from the *omp25* start codon), previously shown to interact with P-BvrR by EMSA (Rivas-Solano et al., 2022), was amplified by PCR with primers omp25R and omp25F (Supplementary Table 1). The 193-bp labeled probe (0.033 μM) was mixed with P-BvrR (0.4 μM) and an excess (1,000×) of each nine ~40 pb unlabeled oligonucleotide pairs obtained by chemical synthesis (Supplementary Table 2), encompassing the region covered by the 193-bp probe. The protein-DNA mixtures were incubated as described for direct EMSA, electrophoresed for 1.5 h, and analyzed as described above.

The oligonucleotide pairs that competed with the 193-bp probe for binding to P-BvrR were then labeled and used as probes in another direct EMSA with increasing concentrations of P-BvrR, as described for the direct EMSA above. Mixtures were electrophoresed for 1 h and analyzed as described above. One of the oligonucleotide pairs that did not compete for P-BvrR was used as a specificity-binding control.

### 2.4. DNase I footprinting

For DNase I footprinting, the same 193-bp region used for competitive EMSA was amplified as described above, labeled with HEX (hexachlorofluorescein) or FAM (5′ 6-carboxyfluorescein), incubated with P-BvrR at 8.32 μM, and proceeded as described for EMSA. The samples were then incubated for 15 min at 15–25°C and placed again in ice. DNAse I (0.05 U) was added, and the samples were incubated for 2 min at 15°C in a thermocycler, followed by 10-min incubation at 75°C. The samples were purified using the Qiagen Qiaquick PCR purification kit and eluted with 30 μl of EB buffer. The samples (10 μl) were run in a 3730 Genetic Analyzer after mixing with 7 μl of HiDi formamide and 0.1 μl of GeneScan 500 Liz size marker and the following running parameters: genotyping module, injection time: 30 s, and injection voltage: 3 kV (Zianni et al., 2006). The Peak Scanner software was used to infer the protected regions by superimposing the electropherograms from digested DNA in the presence of P-BvrR or BSA (3.64 nM). Base pair coordinates of the protected regions were inferred after Sanger sequencing of the 193-bp DNA fragment.

### 2.5. Fluorescence anisotropy

The fluorescence anisotropy assays were performed as described (Owen and McMurray, 2009) with modifications. Recombinant BvrR was phosphorylated as described for EMSA and serially diluted in a binding buffer (10 mM Tris, 1 mM EDTA, 0.1 M NaCl). The forward oligonucleotide 2 (173.133omp25-O, Supplementary Table 2) was 5′-labeled with FAM and mixed with the non-labeled reverse complementary oligo (173.133omp25-ORC, Supplementary Table 2) at a final concentration of 50 mM. The oligonucleotides Oligo rplL-O (5′-FAM labeled) and the reverse complementary Oligo rplL-ORC (Supplementary Table 2) were used as a negative control at a final concentration of 50 mM. Blank samples without protein were also prepared for background fluorescence estimation. Samples were incubated for 30 min at 37°C inside a CytationTM 3 microplate reader (Biotek, Instruments). Fluorescence anisotropy was measured with the appropriate polarized filters, and the results were graphed following a one-site-specific binding model (Favicchio et al., 2009) using the GraphPad Prism (Hulme and Trevethick, 2010; Graph Pad, 2019).

### 2.6. Molecular docking analysis of BvrR-DNA interactions

The interactions between BvrR and its three binding sites were explored by molecular docking using the HDOCK server (default parameters) (Yan et al., 2017). The Fasta BvrR sequence (UniProt accession: Q2YQY4) was used as an input receptor molecule, and the sequences Box 1 (TTGTGTAAGGAGAATGCCAT), Box 2 (GATA TGTCACCCCTGTCAGCGCGG), and Box 3 (CTCGACAGAT TATCTCCACACAATGGGGCA) were used as input ligand molecules. Before the free docking, the software selected the crystal structure of the OmpR-like response regulator KdpE from *E. coli* (RCSB PDB: 4KFC) as a modeling template for the BvrR structure (Seq_ID % = 29.4). To ensure the reliability of the model generated by HDOCK, its quality was analyzed using the QMEANDisCo parameter (Studer et al., 2020) and Ramachandran plots. The model was compared to those generated by SWISS-MODEL (Waterhouse et al., 2018), I-TASSER (Yang et al., 2015), and AlphaFOLD (Jumper et al., 2021; Varadi et al., 2022). The crystal structures of two proteins with DNA-binding domains were used as positive controls for the docking experiments: a *B. abortus* DNA binding protein (RCSB PDB: 4QPJ) and the KdpE protein from *E. coli*. As negative controls, the crystal structures of two proteins lacking DNA-binding domains were used: a *B. suis* 1330 hydrolase (RCSB PDB: 6NQ4) and a *B. abortus* peptidoglycan hydrolase inhibitor (RCSB PDB: 7DPY). For the interpretation, docking scores lower than −200 and confidence scores superior to 0.7 were considered to have good performance and a high likelihood of binding between the analyzed molecules. The NUCPLOT tool (Luscombe et al., 1997) was used to analyze and visualize a 2D interaction coloring scheme of the HDOCK results.

## 3. Results

### 3.1. A 111-bp long fragment at position −262 to −152 is needed for transcriptional activity

In *B. abortus* 2308 W, the *omp25* upstream intergenic region comprises 401 nucleotides (Suárez-Esquivel et al., 2016). To characterize the minimal promoter region of *omp25*, we constructed three plasmid-borne *omp25*-*lacZ* reporter fusions harboring 392-, 262-, and 151-bp upstream of *omp25*, respectively. All three reporter fusions included the first 127-bp of the *omp25* coding sequence (Figure 1A). The resulting plasmids were introduced into *B. abortus* 2308 W-generating strains B392, B262, and B151. Then, we assayed the β-galactosidase activity of each resulting strain at different time points of the growth curve. We used strain 3aZ, carrying a transcriptional chromosomal fusion P*omp3a*::*lacZ*, as the positive control (Table 1). The strain B392 exhibited similar β-galactosidase activity compared to strain 3aZ, except for the late log phase of the growth curve (Supplementary Figure 1; Supplementary Table 3). The strains B392 and B262 reached a peak of β-galactosidase activity at mid-log phase, between 18 and 22 h of growth, without significant statistical differences along the curve (Figure 1B; Supplementary Table 3). However, strain B151, harboring 111-bp less than B262 (Figure 1A), presented significantly reduced β-galactosidase activity compared to B262 and B392. Yet some basal transcriptional activity was observed in this strain at all time points tested, as compared to the empty vector activity (Supplementary Table 3). Therefore, the *omp25* promoter region is located between coordinates −262 and + 127 from the start codon, and the additional 111-bp region in B262 (−262 to −152), as compared to B151, is needed for wild-type transcriptional levels.

### 3.2. The upstream *omp25* regulatory region displays three BvrR binding sites

**Figure 2:**
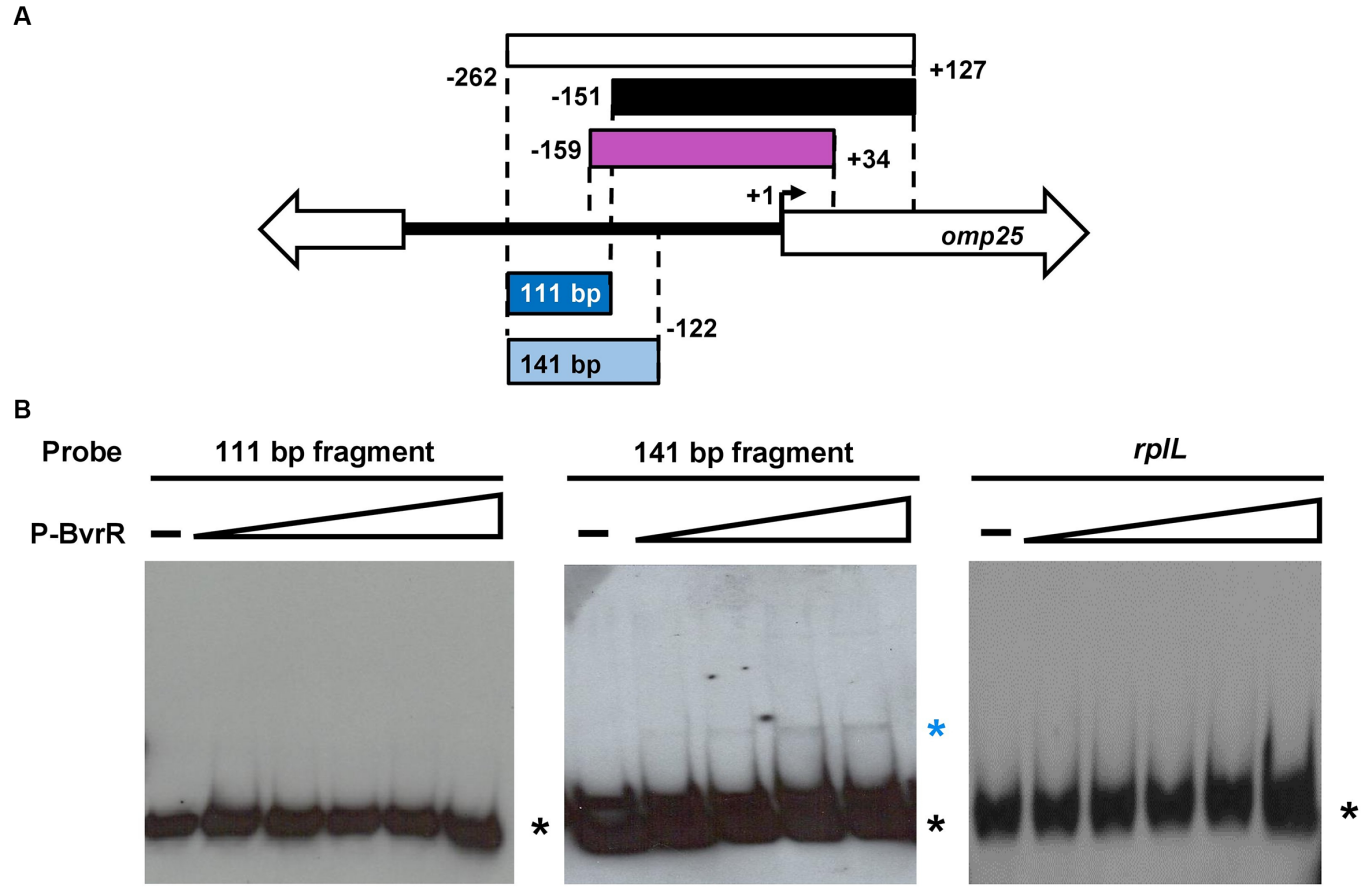
A P-BvrR binding site is inferred by EMSA from −152 to −122. **(A)** Schematic representation of the *omp25* intergenic studied region. White rectangle = fragment displaying wild-type levels of transcription (−262 to +127), black rectangle = fragment displaying basal transcriptional activity (−151 to +127), purple rectangle = fragment previously known to bind to P-BvrR by direct EMSA (−159 to +34), blue rectangle = 111-bp fragment (from −262 to −152) needed to enhance transcription and tested as a probe by direct EMSA with P-BvrR, and light blue rectangle = larger fragment of 141-bp (from −262 to −122), comprising 30 additional downstream bp. **(B)** Direct EMSA results were obtained when using increasing concentrations of P-BvrR from 0.1 to 1.6 μM and one of the following labeled probes: the 111-bp fragment from −262 to −152 (left gel) and the 141-bp fragment from −262 to −122 (middle gel). A 290-bp DNA fragment from the coding region of the ribosomal gene *rplL* (right gel) was used as a specificity P-BvrR binding control. Lanes marked as “-” contain the probe without P-BvrR. Blue asterisks = shifted bands (protein-DNA complexes) selected based on the following criteria: absence in the negative control, P-BvrR concentration dependency, and consistency between independent replicas. Black asterisks = bands with the migration pattern of a free probe. These results are representative of at least three independent experiments.

In *B. abortus* 2308 W, BvrR positively regulates the expression of *omp25* (Guzman-Verri et al., 2002), and a direct binding to the region between −159 and + 34 from the *omp25* start codon has been demonstrated previously (Rivas-Solano et al., 2022). Therefore, based on the results of the β-galactosidase assay, we tested if P-BvrR could also bind by EMSA to the 111-bp fragment required for optimal transcription (−262 to −152, Figure 2A). However, the 111-bp fragment used as a labeled probe and incubated with increasing concentrations of P-BvrR did not reveal shifted bands as compared to the probe alone or to the binding specificity control using *rplL* (Figure 2B), indicating a lack of interaction. Thus, we tested if a larger fragment of 141-bp (−262 to −122, Figure 2A), which included 30 additional bp from the region known to bind to P-BvrR (−159 to +34) (Rivas-Solano et al., 2022), could bind to P-BvrR by direct EMSA. As a result, the probe incubated with growing concentrations of P-BvrR showed shifted bands as compared to the probe alone and the binding specificity control *rplL* (Figure 2B), indicating a specific protein-DNA interaction with the *omp25* upstream region between coordinates −262 and − 122. Altogether, these two direct EMSA results prompted us to infer a putative P-BvrR binding site between positions −151 and − 122 from the *omp25* start codon.

**Figure 3:**
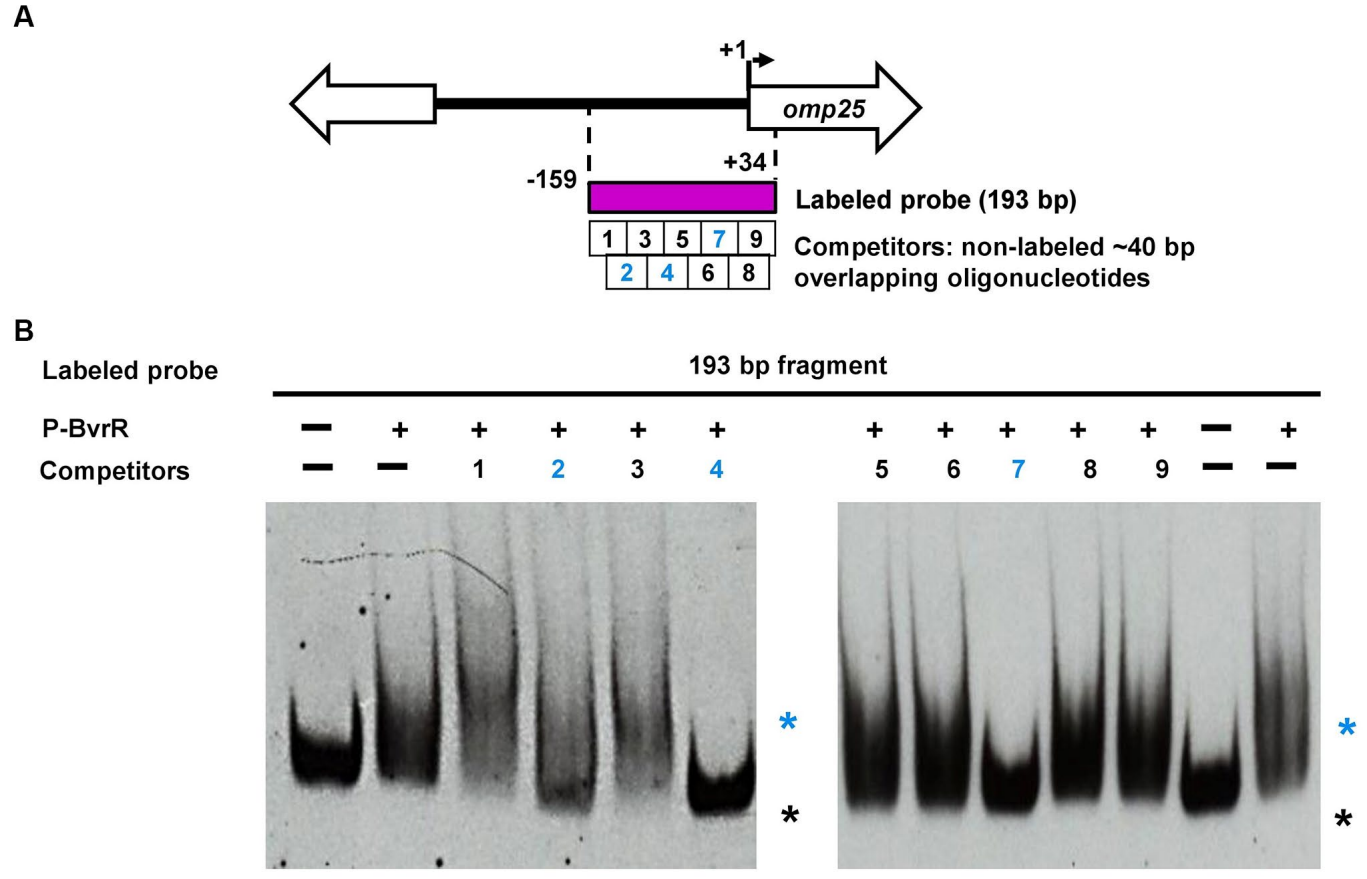
Competitive EMSA results suggest the presence of three putative P-BvrR binding sites on the upstream studied region of *omp25*. **(A)** Schematic representation of the DNA fragments from the *omp25* upstream region that were analyzed in competitive EMSA with P-BvrR. The 193-bp region known to bind to P-BvrR by EMSA (purple rectangle) was used as the labeled probe. This fragment was depicted in nine overlapping 40-bp oligonucleotides that were used as non-labeled competitors (white squares). Numbers in blue = competing oligonucleotides, showing the position of the three putative binding sites. **(B)** Competitive EMSA results, using the 193-bp fragment (−159 to +34) as the labeled probe, P-BvrR (0.4 μM), and an excess (1,000×) of the nine different competitors tested. Lanes marked as “−” contain probes without P-BvrR. Blue asterisks = shifted bands (protein-DNA complexes) selected based on the following criteria: absence in the negative control, P-BvrR concentration dependency, and consistency between independent replicas. Black asterisks = bands with the same migration pattern as a free probe. For oligonucleotides 4 and 7, a clear competition was observed. In the case of oligonucleotide 2, a less defined lower band suggested possible competition. These results are representative of at least three independent experiments.

However, since OmpR has been shown to bind to multiple binding sites on the promoter of its target gene *ompF* (Kenney, 2002), we decided to look for multiple BvrR binding sites in the region from −159 to +34, already known to bind to P-BvrR (Rivas-Solano et al., 2022), including the putatively inferred binding site from −151 to −122. This 193-bp region (−159 to +34) was depicted in nine overlapping sequences of ~40 pb (Figure 3A; Supplementary Table 2). An excess of each non-labeled oligonucleotide was tested in a competitive EMSA with the 193-bp region (−159 to +34) as the labeled probe and P-BvrR at a final concentration of 0.4 μM. As shown in Figure 3B, the oligonucleotides 4 (−100 to −59) and 7 (−39 to +1) outcompeted the 193-bp probe, indicating a specific P-BvrR binding to these oligonucleotides. Additionally, for oligonucleotide 2 (−140 to −100), we observed a less defined lower band that suggested a possible partial competition, although less evident than the one observed for oligonucleotides 4 and 7.

**Figure 4:**
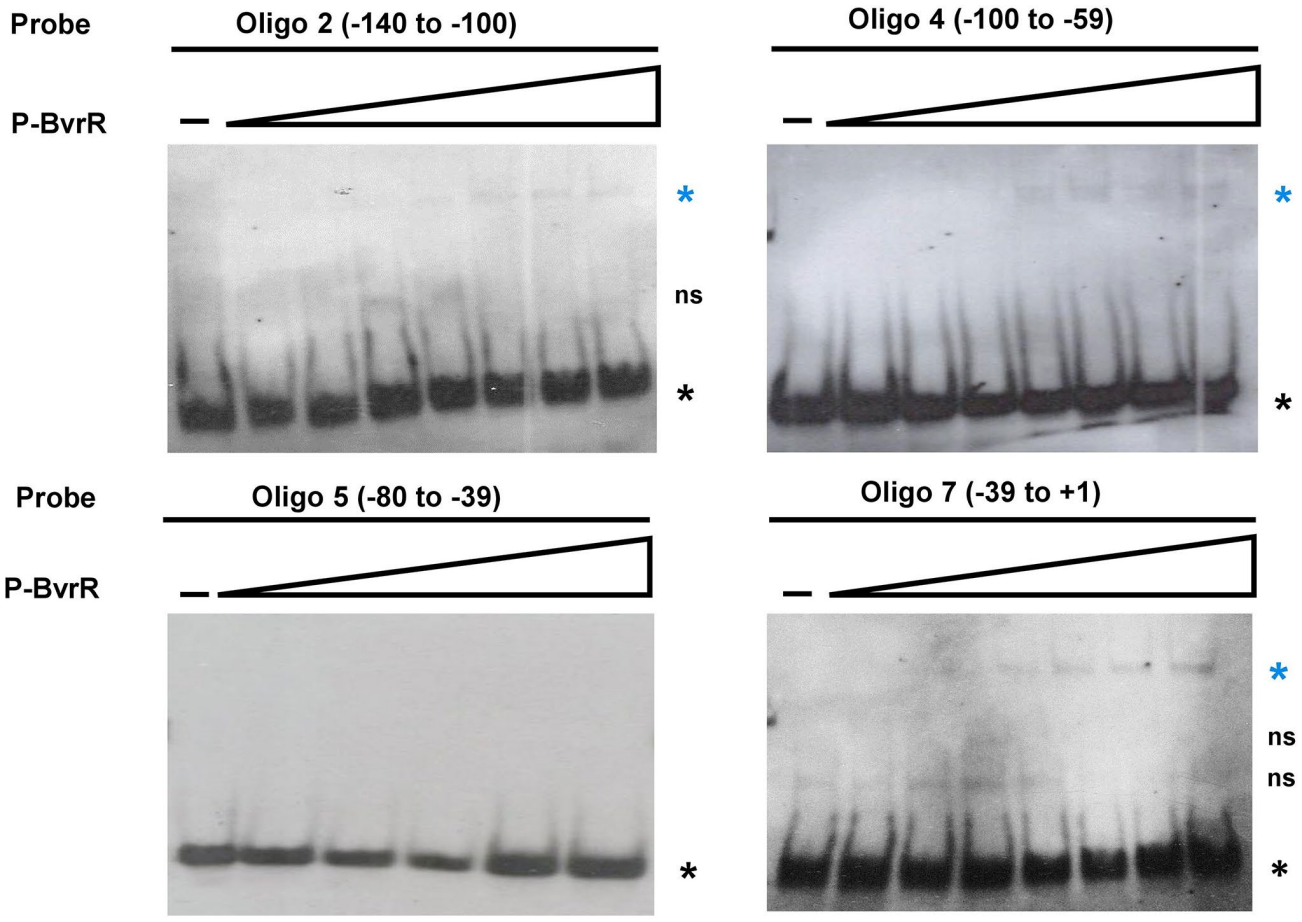
Direct EMSA results confirm the presence of three specific P-BvrR binding sites on the upstream region of *omp25*. The competing oligonucleotides 2, 4, and 7, and the non-competing oligonucleotide 5 (specificity control for P-BvrR binding) were labeled and incubated with increasing concentrations of P-BvrR (0.1 to 1.6 μM). Lanes marked as “-” contain probes without P-BvrR. Blue asterisks = shifted bands (protein-DNA complexes) selected based on the following criteria: absence in the negative control, P-BvrR concentration dependency, and consistency between independent replicas. Black asterisks = bands with the same migration pattern as a free probe. “ns” = non-specific bands. These results are representative of at least three independent experiments.

Subsequently, the oligonucleotides 2, 4, and 7 were labeled to perform a direct EMSA with P-BvrR. We also tested the non-competing oligonucleotide 5 (−80 to −39) as a specificity-binding control. As a result, oligonucleotides 2, 4, and 7 showed shifted bands (Figure 4). We did not observe these interactions with the probe in the absence of P-BvrR and with the specificity binding control (oligonucleotide 5), confirming binding specificity to the three oligonucleotides.

**Figure 5:**
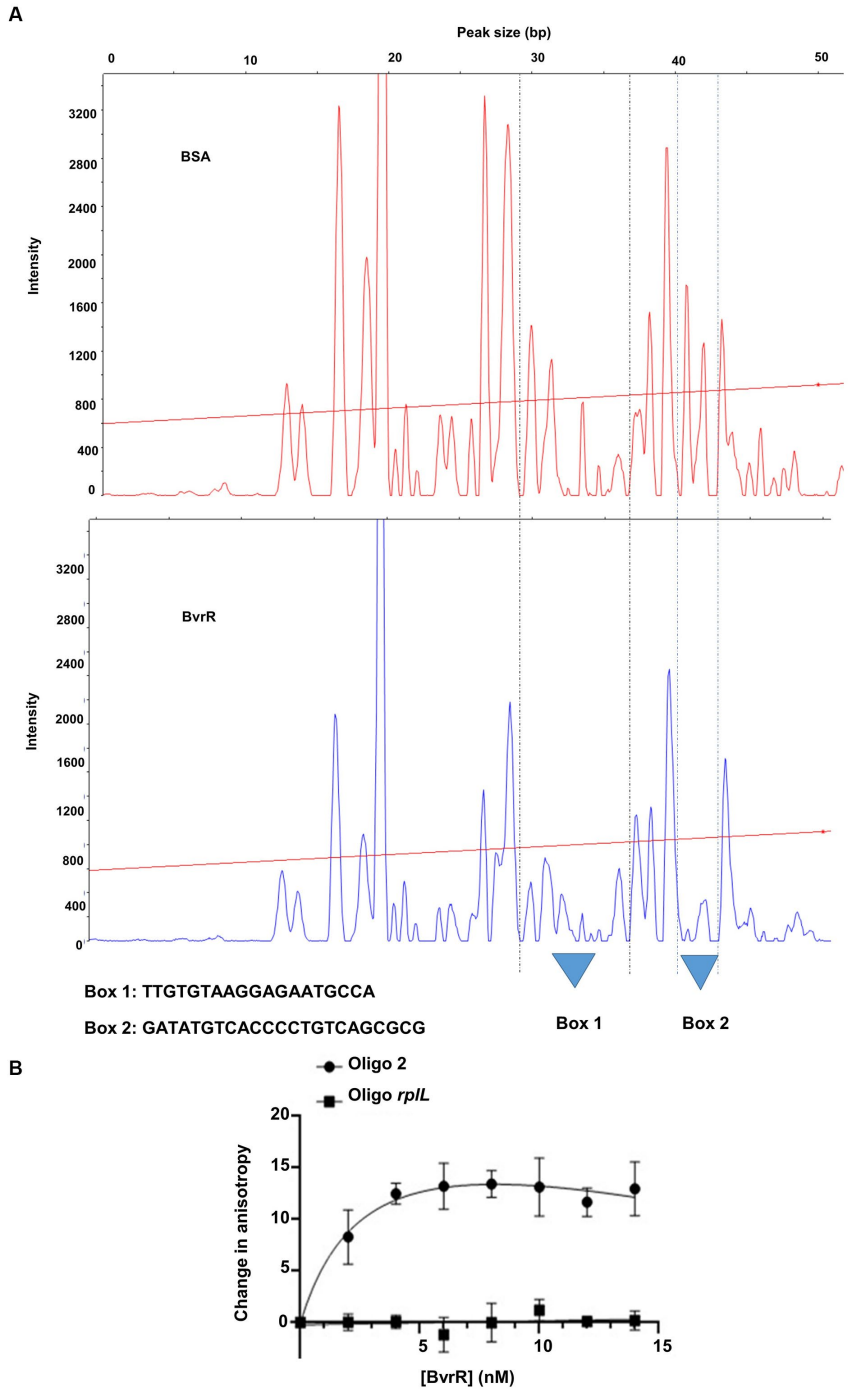
Confirmation of BvrR binding sites by additional experimentation. **(A)** DNase I footprinting analysis of the *omp25* upstream fragment (193-bp, −159 to +34). The Peak Scanner software inferred the protected regions by analyzing the electropherograms from digested DNA with 3.64 nM BSA (upper panel) or 8.32 μM P-BvrR (bottom panel). Base pair positions were inferred from the Sanger sequencing of the 193-bp fragment compared to the DNAse I protected regions. Two protected DNA regions of approximately 20 nucleotides between positions −18 to +1 (box 1) and − 99 to −76 (box 2), matching the oligonucleotides 7 and 4, are shown. The straight red line across each electropherogram represents the molecular size calibration obtained with molecular weight markers run together with the samples. **(B)** Fluorescence anisotropy analysis. The oligonucleotide 2 and a smaller fragment of the negative control used for EMSA were labeled with FAM and separately incubated with P-BvrR. The fluorescence anisotropy of each sample was measured, and the obtained curves show a positive change for the oligonucleotide 2 compared to the negative control. This result, combined with the EMSA results shown in Figures 2–4, allowed us to delimit box 3 to the region between −140 and − 122. These results are representative of at least two independent experiments.

To validate and further delimit the P-BvrR binding sites suggested by the competitive and direct EMSA results, we performed a DNase I footprinting analysis using the entire 193-bp fragment (−159 to +34) and P-BvrR. As a result, we found two protected sequences that we called DNA regulatory boxes: one spanning from −18 to +1 (box 1) and the other from −99 to −76 (box 2) (Figure 5A). Boxes 1 and 2 matched oligonucleotides 7 and 4. However, any clear protected sequence matched oligonucleotide 2 (−140 to −100), which was the one that showed a less clear result in the competitive EMSA (Figure 3B), despite showing binding to P-BvrR in the direct EMSA (Figure 4). To further confirm the binding of P-BvrR to oligonucleotide 2 by another experimental approach, we performed a fluorescence anisotropy assay with 5′-FAM-labeled oligonucleotide 2 and increasing concentrations of P-BvrR. In this method, a fluorescent signal is placed on the smaller DNA molecule, and when it binds to the much larger protein, a change in the fluorescence anisotropy is produced, confirming protein-DNA interactions (Owen and McMurray, 2009). As a BvrR specificity binding control, we included a 5′-FAM-labeled oligonucleotide called oligo *rplL* (Supplementary Table 2). The fluorescence anisotropy results showed a positive change in the anisotropy DNA-binding curve for oligonucleotide 2 compared to the specificity binding control (Figure 5B), confirming P-BvrR-DNA-specific interactions with oligonucleotide 2.

Since oligonucleotide 2 (−140 to −100) partially overlaps the first EMSA-inferred binding site between −151 and − 122, these EMSA and fluorescence anisotropy results allowed us to narrow down this third binding site to a 19-bp region between −140 and − 122, which was named box 3.

#### 3.2.1. Molecular docking and sequence alignment of the three DNA regulatory boxes predict BvrR recognition of inverted and non-inverted DNA repeats

To investigate the theoretical interaction between BvrR and the three DNA regulatory boxes confirmed by different experimental approaches including direct and competitive EMSAs, DNase I footprinting, and fluorescence anisotropy, we performed molecular docking using the HDOCK web server. The quality report of the homology modeling showed that the BvrR model falls within the range of low to medium based on the Seq_ID (Supplementary Table 4). However, based on the TMscore, the model demonstrates high quality (Supplementary Figure 2). Similarly, upon comparing HDOCK’s BvrR model with models generated by SWISS-MODEL, I-TASSER, and AlphaFold using standard quality parameters, we observed only minimal differences among the models (Supplementary Table 5), suggesting that despite its low identity to the template, the model generated by HDOCK shares similarities with those produced by other widely used software.

The three sequences and the positive controls achieved docking scores lower than −200 (Supplementary Table 6), indicating good performance. Furthermore, BvrR and the positive controls exhibited confidence scores superior to 0.7, suggesting a high likelihood of binding between the molecules, while the negative controls scored more than −200 and their confidence scores were lower than 0.7. These results increase the confidence in the binding of BvrR to its ligands.

**Figure 6:**
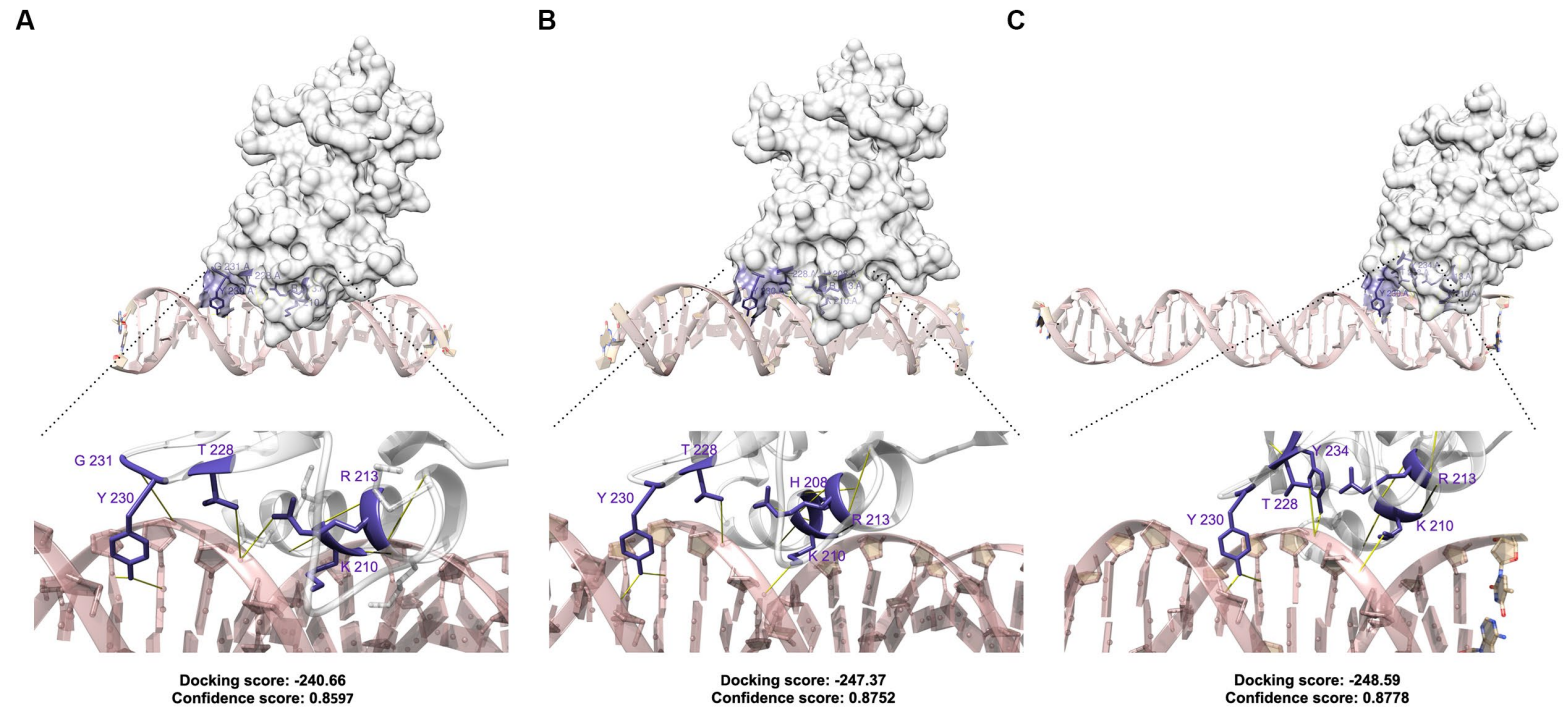
Molecular docking of BvrR and its three binding sites on the promoter region of *omp25.* **(A)** Box 1; **(B)** box 2; and **(C)** box 3. The model of BvrR and the docking with its ligands were generated using the HDOCK server. For the protein, the amino acids involved in hydrogen bonds are highlighted in purple. Hydrogen bridges are represented in yellow.

The docking results predicted the possibility of hydrogen bonds and non-bonded contacts between 13, 15, and 14 amino acid residues from the C terminal domain (Trans_reg_C) of BvrR and DNA portions from boxes 1 (GTAAGGAGAAT), 2 (ACCCCTGTCA), and 3 (AATGGGGC), respectively, with distances in the appropriate range for these bonds (Figure 6 and Supplementary Figure 3). Regarding the amino acids interacting with DNA, in all three boxes, BvrR utilizes the polar and positively charged amino acids Tyr 230, Thr 228, Arg 213, Lys 210, and Tyr 234 to interact with the DNA molecule through hydrogen bonds. When comparing BvrR with the KdpE template, it becomes apparent that the *E. coli* protein primarily utilizes the Tyr, Ile, Gln, and Arg residues to interact with DNA. However, it is worth noting that the DNA interaction site (TTTATA) differs between BvrR and KdpE (Narayanan et al., 2014).

**Figure 7:**
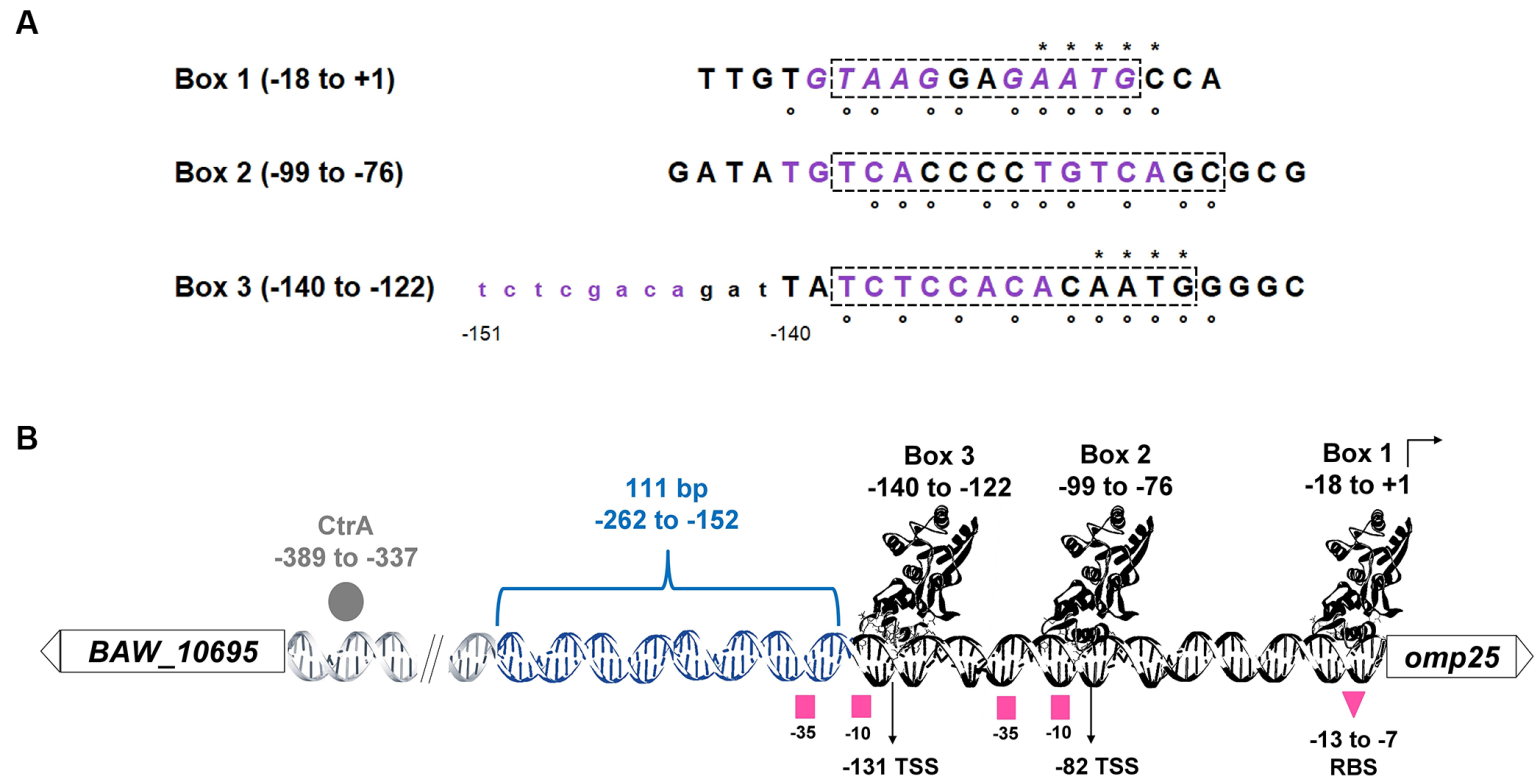
*B. abortus* BvrR regulatory boxes on the regulatory region of *omp25*. **(A)** Nucleotide sequence alignment of the three BvrR regulatory boxes. Dashed squares = nucleotides docked with BvrR. Purple nucleotides = DNA repeats (in italics, the inverted ones in box 1). The 12 upstream nucleotides in lowercase letters next to box 3 (from −151 to −141) were added to show the presence of the repeat. Asterisks = nucleotides matching a 6-nucleotide-long DNA binding motif predicted *in silico* as putatively recognized by BvrR (Ramírez-González et al., 2019). White circles = nucleotides matching a 14-nucleotide-long consensus sequence predicted and experimentally validated for BvrR (Rivas-Solano et al., 2022). **(B)** Non-scale schematic representation of the *omp25* regulatory region. White rectangles = genes *BAW_10695* and *omp25*; DNA chain = intergenic region. Gray circle = CtrA bound to the region from −389 to −337 (Francis et al., 2017), blue DNA chain = 111-bp region from −262 to −152 needed for transcriptional activity, black DNA chain = significantly reduced transcriptional activity, protein structures cartoons = BvrR bound to its three regulatory boxes, right angle arrow = *omp25* coding region, pink squares = predicted −10 and − 35 regulatory elements, pink triangle = predicted ribosome binding site (RBS), vertical black arrows = transcriptional start sites reported elsewhere at positions −131 and − 82, respectively, in bacteria grown until stationary phase (Suárez-Esquivel et al., 2016) and until mid-log phase (Rivas-Solano et al., 2022). The P-BvrR ChIP-Seq signals previously reported have the following coordinates: −242 to −56, −181 to −40, and −159 to +34 (Rivas-Solano et al., 2022).

The sequence alignment of the three boxes (Figure 7A) revealed the presence of the inverted DNA repeat GTAAG – GAATG, separated by two nucleotides (GA) in box 1. In box 2, the non-inverted DNA repeat TGTCA – TGTCA is separated by four nucleotides (CCCC), and nearby in box 3, we found the non-inverted repeat TCTCNACA – TCTCNACA (where N = G or C), separated by five nucleotides (GATTA). Moreover, the sequences of boxes 1 and 3 partially match (83.33 and 66.67%, respectively) a 6-nucleotide long DNA binding motif predicted *in silico* as putatively recognized by BvrR (Ramírez-González et al., 2019). The location of the three boxes is also in agreement with previous P-BvrR ChIP-Seq data, suggesting P-BvrR binding upstream of *omp25* (Rivas-Solano et al., 2022). Since the P-BvrR ChIP-Seq data was used to infer a P-BvrR consensus sequence, it was expected that this region would have some similarity to this sequence. In fact, the three boxes show 78.57 and 71.42% similarity to the 14-bp long consensus sequence (Rivas-Solano et al., 2022).

As shown in Figure 7B, we manually predicted the −35 and −10 elements and the ribosome binding site according to the canonical *E. coli* models. The −35 elements possibly have the sequence GCATTT. This sequence is located at positions −35 to −30 from the first *omp25* TSS, which was described at position −131 from the start codon (Suárez-Esquivel et al., 2016). The sequence GCATTT is also located at positions −41 to −36 from the second TSS described at position −82 from the *omp25* start codon (Rivas-Solano et al., 2022). The −10 elements may have a TATNTC sequence (where N = C or G) located between −10 and − 6 and − 15 and − 10 from the −131 and − 82 TSSs, respectively. The ribosome binding sequence is probably TAAGGAG, located at −13 to −7 from the *omp25* start codon. Detailed sequence information is presented in Supplementary Figure 4.

## 4. Discussion

Despite the crucial role of the TCS BvrR/BvrS in *Brucella*, the DNA-regulatory regions controlled by this TCS are not characterized. Here, we delimited a DNA regulatory region for the gene *omp25*, encoding an outer-membrane protein in *B. abortus* and positively regulated by the TCS BvrR/BvrS (Guzman-Verri et al., 2002). Our results show that a DNA fragment of 380-bp, including 127-bp from the coding region and the first 262-bp upstream of the *omp25* start codon, allows transcription. Additionally, a 111-bp sequence between – 262 and –151 is required for optimal transcription. P-BvrR binds downstream of this 111-bp sequence in three different boxes. It is possible that binding of P-BvrR to these boxes could recruit other transcription factors to impact *omp25* transcription. Many transcription factors play an architectural role in the genome and remodel DNA structure through bending, kinking, wrapping, or bridging (Dorman et al., 2020). In brucellae, other DNA regulatory regions interplay with different transcription factors (de Jong et al., 2008; Sieira, 2013). For instance, the regulatory region of the *virB* operon displays a complex architecture with binding sites for up to six different types of transcriptional regulators, including BvrR, demonstrating a high versatility in responding to various environmental signals at different stages of the infection process (Sieira, 2013). Small RNAs also seem to influence the expression of the *virB* operon in *B. abortus* at a post-transcriptional level (Caswell et al., 2012). Likewise, the regulatory region of *btaE*, a gene encoding a trimeric autotransporter adhesin relevant for virulence (Ruiz-Ranwez et al., 2013), contains binding sites for three different transcription factors also involved in regulating the expression of the *virB* promoter (Sieira et al., 2017). Thus, it seems possible that other transcription factors could work with BvrR to regulate *omp25* expression. In *B. abortus*, the cell-cycle regulator CtrA, conserved in the *Alphaproteobacteria*, has been implicated in controlling outer membrane composition, particularly the abundance and spatial distribution of Omp25 (Francis et al., 2017; Poncin et al., 2018). The CtrA binding site in the *omp25* upstream region is between positions −389 and − 337 (Francis et al., 2017; Figure 7B). Additional transcriptional regulatory mechanisms involved in a BvrR-CtrA interplay remain elusive. In brucellae, mutants in the transcriptional regulators VjbR and GntR display decreased production of Omp25 and altered outer membrane composition (Uzureau et al., 2007; Li et al., 2017). However, the direct interaction between these transcriptional regulators and the regulatory region of *omp25* is currently unknown.

The positions of the BvrR regulatory boxes described here disagree with the canonical *E. coli* models for positive transcriptional regulation. Box 1 (−18 to +1) is next to the *omp25* annotated first codon (Suárez-Esquivel et al., 2016), and box 3 (−140 to −122) includes the transcriptional start site reported for *omp25* at position −131 in brucellae grown to the stationary phase (Suárez-Esquivel et al., 2016), close to −35 and − 10 elements. Additionally, another downstream transcription start site at position −82, matching box 2 (−99 to −76) in brucellae grown to the mid-log phase, has recently been reported (Rivas-Solano et al., 2022). How these regions interact deserves further studies. In prokaryotes, a few transcriptional activators are known to bind to unusual regions to induce promoter activity. For example, in *Bacillus subtilis*, PhoP, a response regulator for phosphate starvation response, induces the activation of the gene *pstS* by binding to an upstream region (−40 to −132) and a coding region (+17 to +270) (Liu et al., 1998). The coding region-box has a low affinity for PhoP-P (Liu et al., 1998), suggesting a dynamic DNA-protein binding in which the regulator is required to start transcription but can easily unbind to allow RNA polymerase to proceed. Global regulators like BvrR can bind to a collection of sites, so the regulatory effect on each binding site would depend on the protein concentration and its affinity. Thus, they could have dual roles as activators, repressors, or both (Martínez-Antonio and Collado-Vides, 2003; Lozada-Chávez et al., 2008; Balleza et al., 2009). The *E. coli* global response regulator OmpR regulates the expression of the gene *ompF* by binding to four sites with different affinities. At low osmolarity, OmpR concentration is low, and the regulator binds to the four boxes, which promotes OmpF expression. At high osmolarity, OmpR concentration increases, and new interactions of OmpR with a distant box (−380 to −350) repress *ompF* transcription (Huang et al., 1994; Kenney, 2002).

Although hydrogen bonds may vary *in vivo*, hydrogen bond formation capability, the docking scores, and the protein-DNA binding position suggest that BvrR has a binding affinity for the three boxes in the Trans_reg_C domain. The DNA-sequence alignment of the three boxes revealed the presence of inverted and non-inverted repeats separated by variable distances, suggesting that variations in the recognition sequences may influence BvrR affinity for a differential regulation of its target genes. The effector domains of some OmpR-like regulators are known to bind tandem sequences or, more rarely, to inverted repeats for the regulation of transcription. The recognition site in the DNA ranges from 18 to 23-bp, with binding sites between 6 and 10-bp separated by 2 to 5-bp of the intervening sequence (Harlocker et al., 1995; Blanco et al., 2002; He and Wang, 2014). However, the regulator ChvI from *S. meliloti* recognizes an 11-bp-long motif sequence present at least once in the analyzed sequences (Chen et al., 2009). Therefore, the target promoters of the OmpR-like response regulators contain multiple binding sites that vary in nucleotide frequency, position, and relative binding affinities. As a result, cooperativity and differential binding are critical components of the transcriptional regulation exerted by the OmpR-like response regulators.

In brucellae, the current model postulates that the TCS BvrR/BvrS senses environmental conditions and regulates gene expression accordingly (Lamontagne et al., 2007; Viadas et al., 2010; Altamirano-Silva et al., 2018, 2021). Based on the results described here, we conclude that (i) A 111-bp region upstream of BvrR binding boxes is needed for wild-type transcriptional levels at different times of the growth curve, suggesting that additional regulators are binding to this region; (ii) P-BvrR could differentially regulate *omp25* expression by direct binding to three DNA regulatory boxes. Whether a particular condition such as phosphorylation or oligomerization affects BvrR binding remains elusive, as well as how many sites are bound simultaneously or independently; and (iii) BvrR possibly recognizes repeated sequences as has been described for other OmpR-like response regulators, and their influence on BvrR binding affinity and preferences remains to be clarified. The oligonucleotides predicted to bind to BvrR by molecular docking could be mutated and tested by EMSA or fluorescence anisotropy with P-BvrR, to prove the impact of each nucleotide on binding affinity. Additionally, crystallography studies of BvrR and BvrR-DNA complexes could also contribute to revealing the mechanistic insights of the binding of BvrR to the regulatory boxes identified here. The results presented here are observations that contribute to a better understanding of the gene regulation mediated by a TCS conserved in *Rhizobiales*, an essential component for environmental adaptation and host–microbe interactions in these organisms. Additional studies should be performed to elucidate the *omp25* transcriptional regulation.

## Data availability statement

The original contributions presented in the study are included in the article/Supplementary material, further inquiries can be directed to the corresponding author.

## Author contributions

AC-Z: conceptualization, formal analysis, investigation, methodology, validation, visualization, roles and writing—original draft, writing—review and editing. OR-S: conceptualization, formal analysis, funding acquisition, investigation, methodology, validation, visualization, roles and writing—original draft, writing—review and editing. FV-R: conceptualization, formal analysis, investigation, methodology, resources, writing—review and editing. OG-E: conceptualization, formal analysis, methodology, resources, writing—review and editing. EM: conceptualization, funding acquisition, supervision, writing—review and editing. EC-O: conceptualization, funding acquisition, resources, supervision, writing—review and editing. CG-V: conceptualization, formal analysis, funding acquisition, investigation, methodology, project administration, resources, supervision, validation, visualization, roles and writing—original draft, writing—review and editing. All authors contributed to the article and approved the submitted version.

## Funding

This study has been funded by grants from “Fondos del Sistema FEES/CONARE” (FS-CN-02-2020 to CG-V), “Fondos FIDA, Universidad Nacional” (SIA 0047-17 to CG-V), and ICGBE (contract CRP/21/005 to CG-V). OR-S holds a Ph.D. scholarship from “Instituto Tecnológico de Costa Rica,” contract number 15-15-D, and from “PINN-MICITT,” contract number PND-137-15-1.

## Conflict of interest

The authors declare that the research was conducted in the absence of any commercial or financial relationships that could be construed as a potential conflict of interest.

## Publisher’s note

All claims expressed in this article are solely those of the authors and do not necessarily represent those of their affiliated organizations, or those of the publisher, the editors and the reviewers. Any product that may be evaluated in this article, or claim that may be made by its manufacturer, is not guaranteed or endorsed by the publisher.

## Supplementary material

The Supplementary material for this article can be found online at: https://www.frontiersin.org/articles/10.3389/fmicb.2023.1241143/full#supplementary-material
