# Supplementary material for: The Brucella abortus two-component system response regulator BvrR binds to three DNA regulatory boxes in the upstream region of omp25
Source: Front Microbiol. 2023 Sep 14;14:1241143. doi: 10.3389/fmicb.2023.1241143 (PMC10538546; doi:10.3389/fmicb.2023.1241143)
Supplement: Supplementary file 1 [file Data_Sheet_1.pdf]

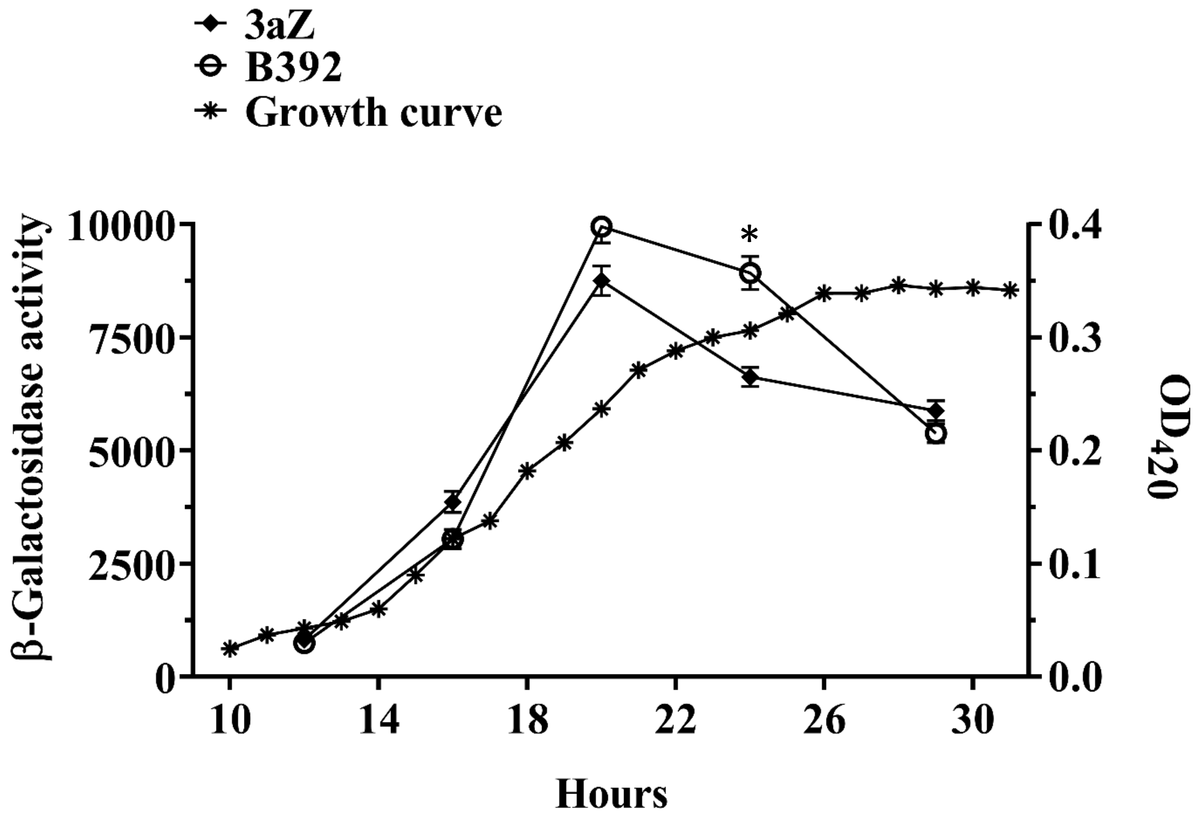


**Supplementary Figure 1.** β-galactosidase activity results for B392 and 3aZ strains. Strain B392 is a *B. abortus* 2308W strain carrying a plasmid-borne *omp25*-*lacZ* reporter fusion harboring 392-bp upstream of *omp25* and the first 127 bp of the coding sequence (B392, unfilled dots). *B. abortus* 2308W 3aZ is a strain carrying a transcriptional chromosomal fusion P*omp3a*::*lacZ* (filled diamond). Both strains were grown in TSB at 37ºC and assayed for β-galactosidase activity at different times of the growth curve. Absorbance was measured at 420 nm at the indicated times. B392 presented significant statistical differences for β-galactosidase activity at late log compared to the 3aZ strain. These results are representative of at least three independent experiments. *, $P <$ 0,05, (One-way ANOVA followed by Turkey´s multiple comparisons test).

**Supplementary Figure 2.** Quality report of homology model by TMalign

Quality report of homology model by TMalign

Template: 4KFC_A.pdb

Seq ID: 29.1%

TMscore: 0.99463

Different ranges of protein homology quality by Seq_ID or TMscore:

| Low | Medium | High |
|---|---|---|
| Seq_ID < 30% | 30% ≤ Seq_ID < 50% | 50% ≤ Seq_ID |
| TMscore < 0.5 | 0.5 ≤ TMscore < 0.8 | 0.8 ≤ TMscore |

**Supplementary Figure 3.** Schematic diagrams of BvrR - Ligands interactions

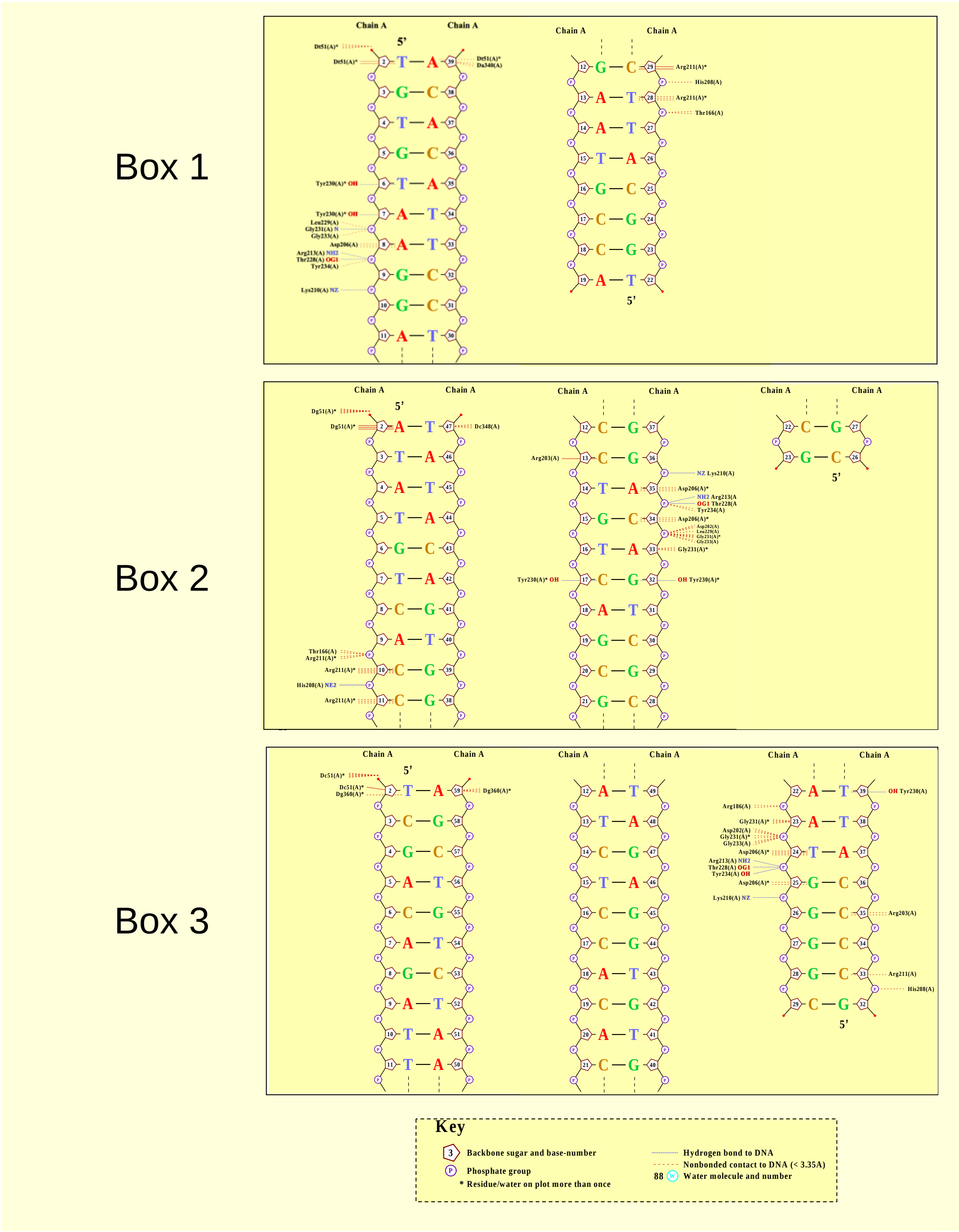

**Supplementary Figure 4.** Complete sequence of *omp25* (BAW_10696) upstream region and coding sequence, integrating the observations generated in this study and other previous relevant information.

tggcaaccggaaaaccatgtctggcgaggtcttcgacgatcgccttcccaatacgccgtgcgccgcctgtcacgaggacggggcaatttg

-392 -389 -337

caaccatgcgggcttcgggatcattcaacacggtaaacgctttcttatcgggattctcaggtcttttcatgccattccggccggaaaaa

-262 -242

agcagcaaaaaatgatgcgagatgcaatgacaactgacaaattatgaaagatttcag**cagcttcgctgcattgctggcaatggtttcggc**

-181 -35 -159 -151

**ttctcctgtctgttgccccttcaatatagggtgtgaaagccggcgttgcgataatgcaacatcgcattttttgccatct**tctcgacagat

-139 / -10 -131 TSS -121 -35 -99 -10 -82 TSS -76 -56

tatctccacAcaatggggcatttcgtgccgcaattaccctcgatatgtcacccctgtcAgcgcggcatgggcggtttactcccgatgct

-40 -18 RBS (-13 to -7) +1 +34

gcccgcccgataagggaccgcgcaaaacgtaatttgtgtaaggagaatgcc***ATG***cgcactcttaagtctctcgtaatcgtctcggctgc

+127

gctgctgccgttctctgcgaccgcttttgctgccgacgccatccaggaacagcctccggttccggctccggttgaagtagctccccagta

tagctgggctggtggctataccggtctttaccttggctatggctggaacaaggccaagaccagcaccgttggcagcatcaagcctgacga

ttggaaggctggcgcctttgctggctggaacttccagcaggaccagatcgtatacggtgttgaaggtgatgcaggttattcctgggccaa

gaagtccaaggacggcctggaagtcaagcagggctttgaaggctcgctgcgtgcccgcgtcggctacgacctgaacccggttatgccgta

cctcacggctggtattgccggttcgcagatcaagcttaacaacggcttggacgacgaaagcaagttccgcgtgggttggacggctggtgc

cggtctcgaagccaagctgacggacaacatcctcggccgcgttgagtaccgttacacccagtacggcaacaagaactatgatctggccgg

tacgactgttcgcaacaagctggacacgcaggatatccgcgtcggcatcggctacaagttctaa

---

-392 to +127 and -262 to +127 = Positive transcriptional activity without statistical differences.

-389 to -337: CtrA binding site (Francis *et al*., 2017).

**-262 to -152**: Region enhancing transcription.

-242 to -56: Region bound to P-BvrR by ChIP-Seq under rich culture conditions (Rivas-Solano *et al*., 2022).

-181 to -40: Region bound to P-BvrR by ChIP-Seq under stress conditions mimicking the intracellular environment (Rivas-Solano *et al*., 2022).

-151 to +127: Basal transcriptional activity.

-159 to +34: Region previously known to bind to P-BvrR by EMSA (Rivas-Solano *et al*., 2022). Region dissected in nine oligonucleotides (40 bp) to EMSA with P-BvrR. Region analyzed by DNase I footprinting with P-BvrR.

-139 to -121: BvrR box 3 delimited by EMSA and fluorescens anisotropy.

tctcnaca: Non-inverted repeat close to box 3, where n = c or g.

-131 (A) and -82 (A): Transcriptional Start Site (TSS) (Suárez-Esquivel *et al*., 2016; Rivas-Solano *et al*., 2022).

gcattt: Predicted -35 element for both TSS.

tatntc: Predicted -10 element for both TSS, where n = c or g.

-99 to -76: BvrR box 2 delimited by DNase I footprinting.

tgtcacccctgtcA: Non inverted repeat separated by two nucleotides in box 2.

-18 to -+1: BvrR box 1 delimited by DNase I footprinting.

gtaag and gaatg: Inverted repeat separated by two nucleotides in box 1.

taaggag: Predicted Ribosome Binding Sequence (RBS) (-13 to -7).

***ATG***: Start codon.

**Supplementary Table 1.** List of primers.

| Primers | Sequence | Size (bp) | Used for |
|---|---|---|---|
| omp25392XbaI<br>omp25.BamHI | GGGATCATTCATCTAGACACACGGTAAACG<br>ACTGGGGAGCGGATCCTACTTCAACC | 521 | β-galactosidase activity |
| omp25.262XbaI<br>omp25.BamHI | CAGCTTCGCTTCTAGAGCATTGCT<br>ACTGGGGAGCGGATCCTACTTCAACC | 391 | β -galactosidase activity |
| omp25.151XbaI<br>omp25.BamHI | TCTCGACAGATTCTAGATATCTCCACACA<br>ACTGGGGAGCGGATCCTACTTCAACC | 280 | β -galactosidase activity |
| omp25lacZF<br>omp25lacZR | ATCCAGGAACAGCCTCCG<br>CCCAGTCACGACGTTGTA | 100 | Promotor fusion screening |
| omp25.262<br>omp25152 | CAGCTTCGCTGCATTGCT<br>AGATGGCAAAAATGCGATG | 111 | EMSA |
| omp25.262<br>omp25.122 | CAGCTTCGCTGCATTGCT<br>GCCCCATTGTGTGGAGATAA | 141 | EMSA |
| omp25F<br>omp25R | GCCATCTTCTCGACAGATTATC<br>GTCTCTCGTAATCGTCTCGG | 193 | DNAaseI footprinting |
| L12.F<br>L12.R | GGCTGATCTCGCAAAGAT<br>CCAGGTCCTTGGCTTCCTTGAG | 290 | Specificity binding control for EMSA |

The underline bases correspond to the target of the restriction enzymes: TCTAGA for XbaI and GGATCC for BamHI

**Supplementary Table 2.** List of oligonucleotides used for EMSA or fluorescence anisotropy and their position in *omp25* upstream region.

| Number | Oligonucleotide* | Sequence | Position |
|---|---|---|---|
| 1 | 193.153omp25-O | GCCATCTTCTCGACAGATTATCTCCACACAATGGGGCATT | -159 to -120 |
| | 193.153omp25-ORC | AATGCCCCATTGTGTGGAGATAATCTGTCGAGAAGATGGC | |
| 2 | 173.133omp25-O | ATCTCCACACAATGGGGCATTTCGTGCCGCAATTACCCTCG | -140 to -100 |
| | 173.133omp25-ORC | CGAGGGTAATTGCGGCACGAAATGCCCCATTGTGTGGAGAT | |
| 3 | 153.113omp25-O | TTCGTGCCGCAATTACCCTCGATATGTCACCCCTGTCAGCG | -120 to -80 |
| | 153.113omp25-ORC | CGCTGACAGGGGTGACATATCGAGGGTAATTGCGGCACGAA | |
| 4 | 133.93omp25-O | GATATGTCACCCCTGTCAGCGCGGCATGGGCGGTTTACTCC | -100 to -59 |
| | 133.93omp25-ORC | GGAGTAAACCGCCCATGCCGCGCTGACAGGGGTGACATATC | |
| 5 | 113.73omp25-O | GCGGCATGGGCGGTTTACTCCCGATGCTGCCCGCCCGATAA | -80 to -39 |
| | 113.73omp25-ORC | GCGGCATGGGCGGTTTACTCCCGATGCTGCCCGCCCGATAA | |
| 6 | 93.53omp25-O | CCGATGCTGCCCGCCCGATAAGGGACCGCGCAAAACGTAAT | -59 to -19 |
| | 93.53omp25-ORC | ATTACGTTTTGCGCGGTCCCTTATCGGGCGGGCAGCATCGG | |
| 7 | 73.33omp25-O | AGGGACCGCGCAAAACGTAATTTGTGTAAGGAGAATGCCA | -39 to +1 |
| | 73.33omp25-ORC | TGGCATTCTCCTTACACAAATTACGTTTTGCGCGGTCCCT | |
| 8 | 53.13omp25-O | TTTGTGTAAGGAGAATGCCATGCGCACTCTTAAGTCTCTCG | -19 to +22 |
| | 53.13omp25-ORC | CGAGAGACTTAAGAGTGCGCATGGCATTCTCCTTACACAAA | |
| 9 | 33.00omp25-O | ATGCGCACTCTTAAGTCTCTCGTAATCGTCTCGG | +1 to +34 |
| | 33.00omp25-ORC | CCGAGACGATTACGAGAGACTTAAGAGTGCGCAT | |
| -** | Oligo rplL-O | CCTTTCGGCCCTGACCGTTCTGGAAGCCGCTGAGCTGTCC | N.A.*** |
| | Oligo rplL-ORC | GGACAGCTCAGCGGCTTCCAGAACGGTCAGGGCCGAAAGG | |

* “O” = forward, “ORC” = reverse complementary.

** “-” = BvrR specificity binding control used for fluorescence anisotropy.

*** “N.A.” = not applicable = smaller fragment from the BvrR specificity binding control used for EMSA (gene *rplL*).

**Supplementary Table 3.** Miller Units means for each of the strains assayed.

| | Miller Units mean | | | | | | | | | |
|---|---|---|---|---|---|---|---|---|---|---|
| **Growth curve phase/Strain** | **3aZ** | SD | **B392** | SD | **B262** | SD | **B151** | SD | **BpMR15** | SD |
| Lag | 822.8 | 135.1 | 751.6 | 123.7 | 676.3 | 159.5 | 179.4 * | 63.3 | 0.0 | 0.0 |
| Early log | 3864.0 | 233.0 | 3048.0 | 213.9 | 2659.0 | 174.2 | 165.6 * | 33.9 | 0.0 | 0.0 |
| Mid log | 8750.0 | 326.7 | 9947.0 | 361.2 | 9094.0 | 448.8 | 187.0 * | 11.1 | 84.7 | 17.0 |
| Late log | 6625.0 * | 211.3 | 8921.0 | 368.7 | 7824.0 | 202.9 | 224.4 * | 42.5 | 28.8 | 10.9 |
| Stationary | 5882.0 | 224.5 | 5380.0 | 206.6 | 5262.0 | 242.1 | 195.0 * | 12.9 | 138.1 | 6.3 |

These results are the means of at least three independent experiments. The means for the strains 3aZ, B392, B262 and B151 are the values obtained after subtracting the mean for the strain with the empty vector (BpMR15). These values were plotted in figure 1B and supplementary figure 1. Significant statistical differences were obtained by one-way ANOVA statistical analysis followed by Turkey´s multiple comparisons test using GraphPad Prism version 8.00 for Windows (GraphPad Software, La Jolla, California, USA).

SD: Standard Deviation.

* Statistically significant different means between this value and any other value at the same growth curve phase, except for the empty vector containing strain (BpMR15) ($P < 0.05$).

**Supplementary Table 4.** Complex Template Information.

| Molecule | PDB ID | Chain | Align_length | Coverage | Seq_ID (%) |
|---|---|---|---|---|---|
| Receptor (BvrR) | 4KFC | A | 231 | 0.967 | 29.4 |

**Supplementary Table 5.** Structure validation and evaluation: overall value of QMEANDisCo and Ramachandran plot review for different homology-modeled 3D BvrR structures.

| Parameter | Modeling strategy | | | |
|---|---|---|---|---|
| | HDOCK (MODELLER) | SWISS-MODEL | I-TASSER | AlphaFOLD |
| QMEANDisCo ± 0.05 | 0.65 | 0.63 | 0.65 | 0.69 |
| Residues in most favoured regions | 193 | 190 | 169 | 200 |
| Residues in additional allowed regions | 14 | 18 | 41 | 15 |
| Residues in generously allowed regions | 2 | 1 | 4 | 0 |
| Residues in disallowed regions | 1 | 2 | 2 | 1 |

**Supplementary Table 6.** Docking score and Confidence score of the docking interactions between the BvrR, positive and negative controls with the three ligands.

| | Docking score / Confidence score | | | | |
|---|---|---|---|---|---|
| | BvrR | Positive control 1 (4KFC) | Positive control 2 (4QPJ) | Negative control 1 (7DPY) | Negative control 2 (6nq4) |
| Box 1 | -240.66 / 0.8597 | -241.10 / 0.8608 | -214.13 / 0.7829 | -173.72/ 0.6164 | -180.72 / 0.6489 |
| Box 2 | -247.37 / 0.8752 | -229.70 / 0.8312 | -204.25 / 0.7474 | -187.85 / 0.6807 | -175.94 / 0.6269 |
| Box 3 | -248.59 / 0.8778 | -245.62 / 0.8713 | -213.82 / 0.7818 | -175.14 / 0.6231 | -175.51 / 0.6249 |
